# Declining Procedures by Pediatric Critical Care Medicine Fellowship Trainees

**DOI:** 10.3389/fped.2018.00365

**Published:** 2018-11-30

**Authors:** Branden M. Engorn, Christopher J. L. Newth, Margaret J. Klein, Elizabeth A. Bragg, Rebecca D. Margolis, Patrick A. Ross

**Affiliations:** ^1^Department of Anesthesiology Critical Care Medicine, Children's Hospital Los Angeles, Keck School of Medicine of University of Southern California, Los Angeles, CA, United States; ^2^Departments of Anesthesiology Critical Care Medicine and Pediatrics, Children's Hospital Los Angeles, Keck School of Medicine of University of Southern California, Los Angeles, CA, United States; ^3^Department of Anesthesiology Critical Care Medicine, Children's Hospital Los Angeles, Los Angeles, CA, United States

**Keywords:** pediatric critical care, medical education, fellowship, arterial line, central venous access, intubation

## Abstract

**Background:** Pediatric Critical Care Medicine Fellowship trainees need to acquire skills to perform procedures. Over the last several years there have been advances that allowed for less invasive forms of interventions.

**Objective:** Our hypothesis was that over the past decade the rate of procedures performed by Pediatric Critical Care Medicine Fellowship trainees decreased.

**Methods:** Retrospective review at a single institution, tertiary, academic, children's hospital of patients admitted from July 1, 2007–June 30, 2017 to the Pediatric Intensive Care Unit and Cardiothoracic Intensive Care Unit. A Poisson regression model with a scale adjustment for over-dispersion estimated by the square root of Pearson's Chi-Square/DOF was applied.

**Results:** There has been a statistically significant decrease in the average rate of central venous lines (*p* = 0.004; −5.72; 95% CI: −9.45, −1.82) and arterial lines (*p* = 0.02; −7.8; 95% CI: −13.90, −1.25) per Fellow per years in Fellowship over the last 10 years. There was no difference in the rate of intubations per Fellow per years in Fellowship (*p* = 0.27; 1.86; 95% CI:−1.38, 5.24).

**Conclusions:** There has been a statistically significant decrease in the rate of central venous lines and arterial lines performed by Pediatric Critical Care Medicine Fellowship trainees per number of years in Fellowship over the last 10 years. Educators need to be constantly reassessing the clinical landscape in an effort to make sure that trainees are receiving adequate educational experiences as this has the potential for an impact on the education of trainees and the safety of the patients that they care for.

## Introduction

Pediatric Critical Care Medicine (PCCM) Fellowship trainees need to acquire the skills necessary to provide airway support and vascular access to critically ill patients. The Accreditation of Graduate Medical Education (ACGME) and American Board of Pediatrics (ABP) expect that over 3 years of training, PCCM Fellows become proficient in: arterial and central venous catheterization; endotracheal intubation; and thoracostomy tube placement (1). However, neither the ACGME nor the ABP states a required number of successfully completed procedures to demonstrate competence, but rather relies on the individual Fellowship training program to ensure proficiency. Many Residents are performing few procedures (2, 3) and therefore the subsequent years of Fellowship training are a finite period to obtain competence. Failure to complete a procedure is associated with increased risk of patient safety events (4–7). PCCM and similar medical specialties, such as Anesthesiology and Emergency Medicine, have determined a minimum number of procedures before an expected competency (success for completing a given procedure) for a given procedure would be obtained (8–11).

The purpose of this retrospective study was to review the rate of procedures being performed by PCCM Fellow trainees in a large, tertiary academic children's hospital. Our hypothesis was that over the past 10 years the rate of procedures performed by Fellowship trainees in the Pediatric Intensive Care Unit (PICU) and Cardiothoracic Intensive Care Unit (CTICU) decreased over time at a single, academic institution. Our primary outcome was the rate of procedures per Fellow per year in Fellowship and we broke down the procedures into intubations, central venous lines (CVL), and arterial lines. Secondary outcomes included the median number of intubations, CVL, and arterial lines per Fellow per year.

## Materials and methods

After receiving institutional review board approval (Children's Hospital Los Angeles CHLA-17-00318) we reviewed all cases admitted to the PICU and CTICU from July 1, 2007–June 30, 2017. Our PICU and CTICU maintain a clinical and research database in which patient information including procedures is inserted on a real-time basis by PCCM Fellows and Attendings. Manuscripts using this database have previously been published (12). From this database all children admitted were identified and their charts retrospectively reviewed. Data abstracted included the total number of admissions, percentage of admissions that were medical and surgical, intensive care unit (ICU) length of stay (LOS), and ICU length of mechanical ventilation (LMV). The number of procedures performed was calculated by a count of the number of procedure notes (intubation, CVL, and arterial lines) documented by each Fellow during each academic year. The procedure notes include procedures performed by the Fellow in the PICU, CTICU, Emergency Department, and Inpatient Ward. This count was then collated and summed to include a combined number of individual procedures from both the PICU and CTICU for each Fellow. Pediatric risk of mortality (PRISM III) scores were abstracted for each academic year from our local Virtual Pediatric System database. The Society of Thoracic Surgeons-European Association for Cardio-Thoracic Surgery (STAT) scores were abstracted from our local database. To determine the number of Fellows per year we compared the number of Fellows per year as documented on the ABP roster and compared it with the number of Fellows documenting procedures in a given academic year. We reconciled the total Fellow count to ensure inclusion of both ACMGE accredited PCCM Fellows and non-ACGME accredited CTICU Fellows (for which our institution has 0–2 per academic year). To create the Poisson Regression models each Fellow was assigned their final year as their academic year and the rate of each procedure was calculated by dividing the total number of a given procedure by the number of years in the program. We chose to standardize the procedures performed per Fellow per years to consider Fellows participating in the Fellowship for various number of years depending on factors including completion of a 3-year critical care medicine Fellowship, 1-year CTICU Fellowship, extending Fellowship for personal or professional reasons, terminating Fellowship early, and to include fellows on both extremes of the decade as they finish or start training.

### Outcomes

Primary outcome was the rate of intubations, CVL, and arterial lines performed per Fellow per years in the Fellowship program. Secondary outcomes included the median number of intubations, CVL, and arterial lines per Fellow per year.

### Statistical analysis

Data are expressed as total counts, medians (interquartile range), and percentages. Poisson regression models using a scale adjustment estimated by the square root of Pearson's Chi-Square/DOF for over-dispersion were applied (13). We chose to analyze the data with a Poisson regression to account for the count like nature of the data being skewed to the right. Model estimates originally calculated on the log scale using Poisson regression were exponentiated and converted to a multiplicative percent change for interpretation. For each type of procedure, the outcome was the total number of procedures done during Fellowship per number of years in the Fellowship program, as predicated by the final academic year of the Fellowship. To determine if the rate of procedures were decreasing over time, the effect of time was assessed at a *p* < 0.05 significance level. All statistical analysis was performed with consultation of a biostatistician. Data analysis and output generated using SAS software version 9.4. (Copyright2002–2012 SAS Institute Inc., Cary, NC, USA).

## Results

### Baseline characteristics

Baseline characteristics of the PICU, CTICU, and Fellowship program over the course of 10 years are shown in Table 1. The PICU and CTICU were similar in terms of PRISM score, percentage of patients per STAT category, LOS, and LMV. There was an increase in the percentage of medical patients in both the PICU and CTICU over the course of the decade. The Fellowship program grew in number of Fellows per year over time, but this growth was to follow the increase in clinical volume demonstrated by a similar number of admissions per Fellow over the 10-year study period. The years are listed as academic years (ex. 2017 is July 1, 2016–June 30, 2017).

**Table 1:** Characteristics of the Pediatric Intensive Care Unit, Cardiothoracic Intensive Care Unit, and Critical Care Medicine Fellowship Program.

	**2008**	**2009**	**2010**	**2011**	**2012**	**2013**	**2014**	**2015**	**2016**	**2017**
Total ICU admissions	1,924	1,931	1,855	1,854	2,051	2,116	2,247	2,498	2,558	2,589
PICU admissions	1,223	1,202	1,150	1,186	1,245	1,354	1,415	1,578	1,643	1,616
PICU% medical	52.2	47.8	47.6	46.7	47.6	57.6	67	66.3	71	73.5
PICU% surgical	47.8	52.2	52.4	53.3	52.4	42.4	33	33.7	29	25.7
PICU LOS (days)	3	3	4	4	3	3	4	3	3	3
PICU LMV (days)	1.9	2.4	3.1	2.0	2.7	2.4	2.0	2.5	1.9	2.8
PICU PRISM	UN	3	3	3	3	2	3	3	2	2
CTICU admissions	701	729	705	668	806	762	832	920	915	973
CTICU% medical	36.8	21.4	27.4	18.6	25.9	32	42.7	49.9	40.1	43.6
CTICU% surgical	63.2	78.6	72.6	81.4	74.1	68	57.3	50.1	59.9	56.4
CTICU LOS (days)	4	4	4	4	4	4	4	4	5	4
CTICU LMV (days)	2.2	2.0	2.0	1.5	1.9	2.0	1.8	1.8	1.9	1.8
CTICU %										
STAT 1–3 STAT 4–5 No Score	59.2 28.9 11.9	58.4 26.2 15.4	61.6 21.9 16.5	68.3 20.0 11.6	65.5 23.2 11.4	67.5 22.2 10.3	60.5 25.0 14.4	58.3 28.0 13.7	55.0 28.9 15.1	55.5 28.1 22.6
Fellow count	9	11	11	11	12	13	14	13	13	14
Total admissions/Fellow	213.8	175.6	168.6	168.6	171.0	162.8	160.5	192.1	196.8	184.9
PICU admissions/fellow	135.9	109.3	104.5	107.8	103.8	104.2	101.1	121.4	126.4	115.4
CTICU admissions/fellow	77.9	66.3	64.1	60.7	67.2	58.6	59.4	70.8	70.3	69.5

### Rate of procedures

The Poisson regression model is demonstrated in Table 2. There has been a statistically significant decrease in the average rate of CVL (*p* = 0.004; −5.72; 95% CI: −9.45, −1.82) and arterial lines (*p* = 0.02; −7.8; 95% CI: −13.90, −1.25) performed per Fellow per years in the Fellowship program over the last 10 years. There was no difference in the rate of intubations per Fellow per years in Fellowship (*p* = 0.27; 1.86; 95% CI: −1.38, 5.24) over the last 10 years.

**Table 2:** Poisson Regression Model Estimates.

**Model outcome**	**Estimated effect of time (% change)^1^**	***P*-value**
Total intubations per number of years in fellowship	1.86 (−1.38, 5.24)	0.27
Total central lines per number of years in fellowship	−5.72 (−9.45, −1.82)	0.004
Total arterial lines per number of years in fellowship	−7.80 (−13.90, −1.25)	0.02

1 *Multiplicative effect in terms of percent change with 95% confidence interval; i.e., for every 1 year increase, the estimated rate of total central lines completed during fellowship per number of years in the fellowship program decrease by 5.72%, on average*.

### Intubations, CVL, and arterial lines per fellow per year

**Figure 1:**
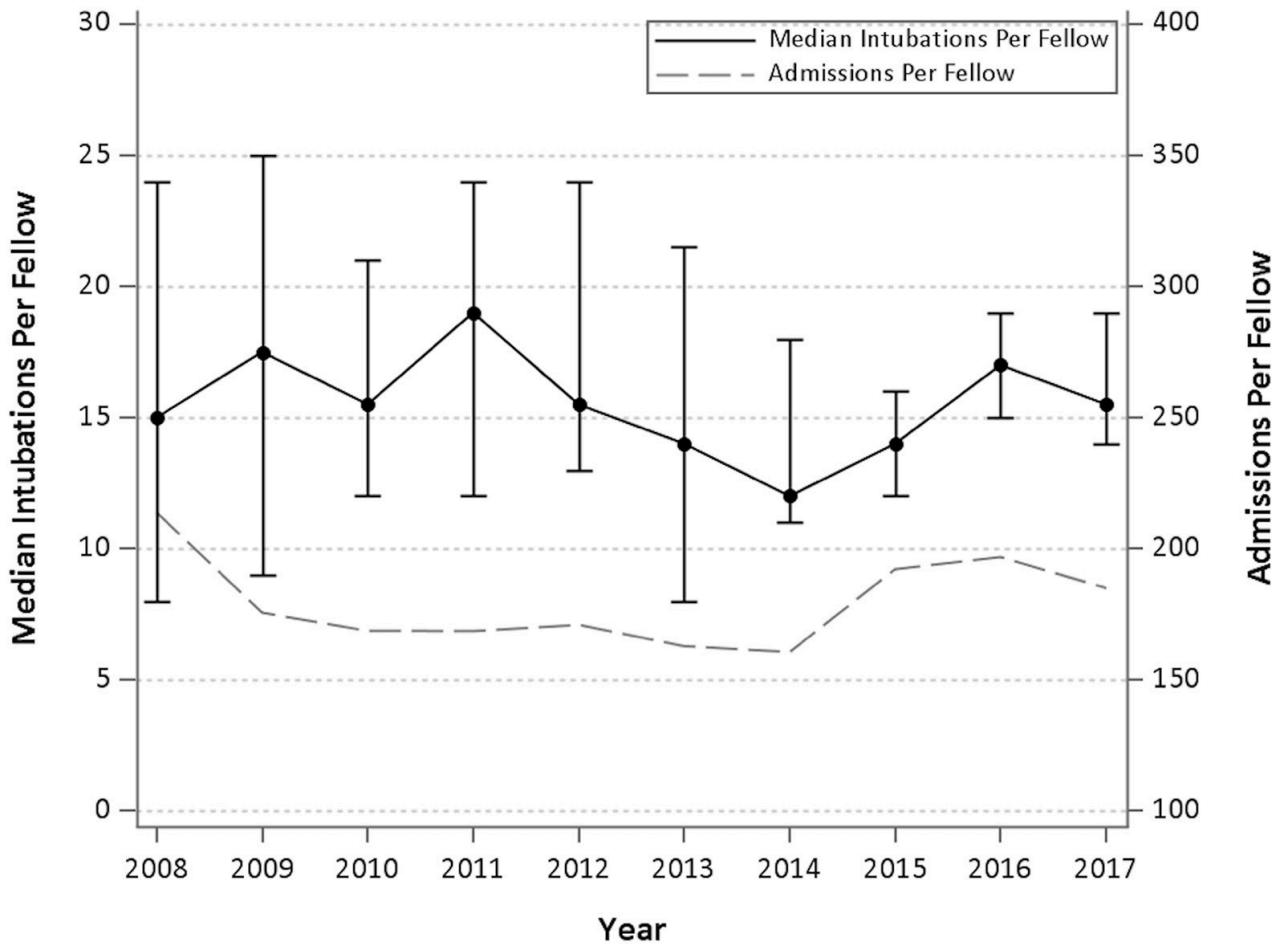
Median and interquartile range of intubations per Fellow per year and admissions per Fellow year over 10 years.

**Figure 2:**
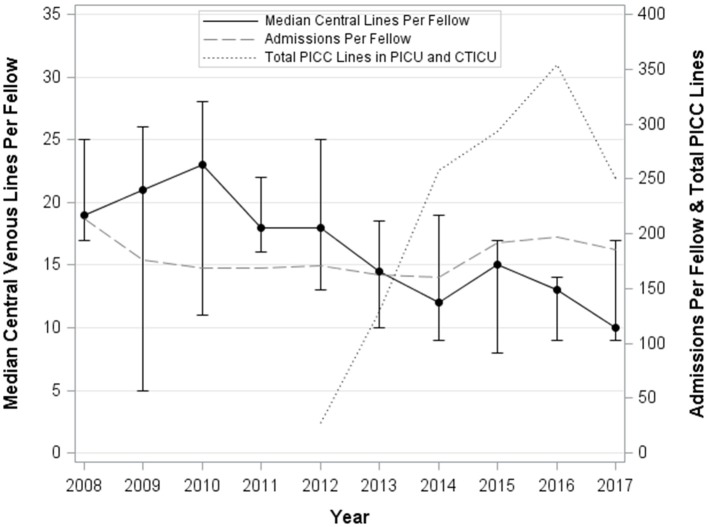
Median and interquartile range of central venous lines per Fellow per year, total nursing PICC lines per year, and admissions per Fellow year over 10 years.

**Figure 3:**
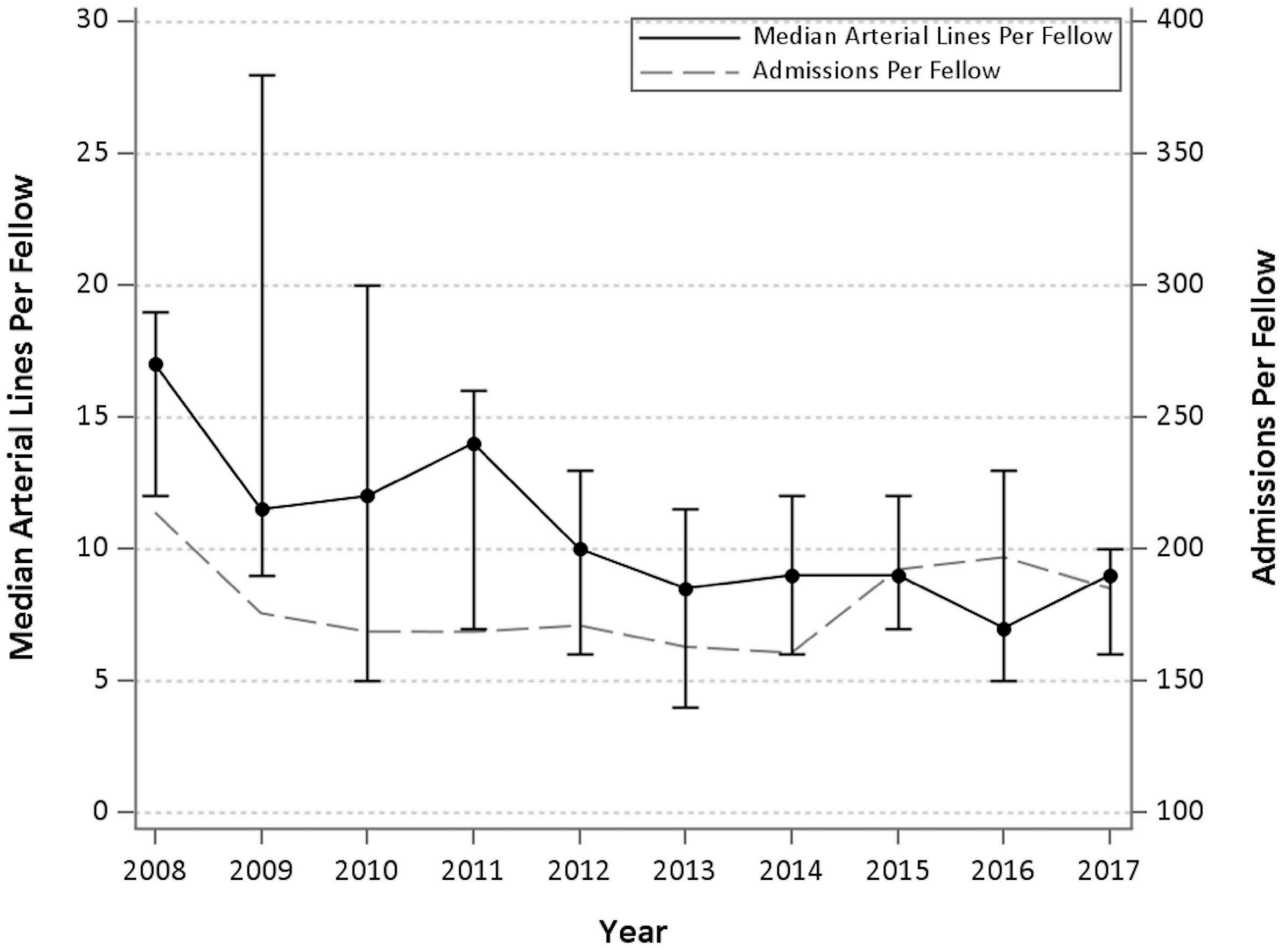
Median and interquartile range of arterial lines per Fellow per year and admissions per Fellow year over 10 years.

The median number of intubations, CVL, and arterial lines per Fellow per year are demonstrated in Table 3. Figures 1–3 graphically shows that median intubations were unchanged over the 10-year study period while both CVL and arterial lines decreased. Figure 2 demonstrates a temporal relationship with an increase in peripherally inserted central catheters (PICC) and decrease in Fellow CVL.

**Table 3:** Absolute count, median and interquartile range per Fellow per year, and Rate per admission of Intubations, Central Venous Lines, and Arterial Lines.

	**2008**	**2009**	**2010**	**2011**	**2012**	**2013**	**2014**	**2015**	**2016**	**2017**
Intubations	144	174	153	199	161	171	183	204	206	217
Median	15	17.5	15.5	19	15.5	14	12	14	17	15.5
1, 3 interquartile range	8, 24	11, 24.3	12, 19.8	13, 24	13, 22.8	8.5, 20.3	11, 18	12, 16	15, 19	14.3, 18.8
Rate per admission (%)	7.5	9.0	8.2	10.7	7.8	8.1	8.1	8.2	8.1	8.4
Central venous lines	189	226	280	211	223	178	183	183	166	167
Median	19	21	23	18	18	14.5	12	15	13	10
1, 3 interquartile range	17, 25	8, 25.5	15, 27.5	16.5, 21.5	14, 24.5	11, 18.3	9, 19	8, 17	9, 14	9, 17
Rate per admission (%)	9.8	11.7	15.1	11.4	10.9	8.4	8.1	7.3	6.5	6.5
Arterial lines	179	220	179	136	133	99	156	131	148	131
Median	17	11.5	12	14	10	8.5	9	9	7	9
1, 3 interquartile range	12, 19	9, 25	5.2, 18.8	9, 15	6.5, 13	4, 10.8	6, 12	7, 12	5, 13	6, 10
Rate per admission (%)	9.3	11.4	9.6	7.3	6.5	4.7	6.9	5.2	5.8	5.1

## Discussion

Our findings suggest that over the last 10 years at a tertiary, academic, children's hospital there has been a statistically significant decrease in the rate of procedures per Fellow per years in Fellowship for CVL and arterial lines but not for intubations.

Advances in Critical Care Medicine have allowed for less invasive forms of procedures. For example, non-invasive ventilation has been shown to decrease the need for endotracheal intubation (14, 15). PICC lines may be decreasing the numbers of CVL being placed in the PICU (16, 17). Intraosseous lines may substitute for CVL for emergent fluid resuscitation (18). Vasoactive agents can be safely administered for short periods of time through a peripheral intravenous line (19) and recent guidelines endorse early vasoactive agents through a peripheral intravenous or intraosseous line in pediatric and neonatal shock (20). Finally, pulse oximetry is likely accurate enough to replace arterial lines for ventilator management in acute respiratory failure through use of the pulse oximetric saturation Spo2/Fio2 (SF) ratio (21–23).

Our data did not demonstrate a decrease in rates of intubation. In medical specialties that perform similar procedures to PCCM, such as Anesthesiology and Emergency Medicine, authors have described the number of intubations required to achieve competence and a mean of 44–74 tracheal intubations achieved 80–90% competency within one-two attempts (8–10). During PCCM Fellowship training a median of 26 tracheal intubations outside of the operating room were needed to achieve 90% competency within four attempts (11). However, in allowing for up to four attempts there are likely an increased patient safety events in pediatric patients (4, 7). Finally, while we saw no decline in intubations performed over the past decade the number performed is still less than was experienced in our institution in the past. In review of our Fellow's procedure logs from 1994 the average number of admissions per fellow was 108 and number of intubations was 44 intubations per Fellow per year (i.e., triple the current rate). However, we recognize this occurred in a time period with different work hours and presentation of pediatric diseases.

Our CVL data showed a decreasing rate over time. At our institution, there has been a dedicated PICC nursing team since 2012. Figure 2 demonstrates that with the increase in nursing placed PICC lines there has been a temporal relationship with a decrease in Fellow CVL. We suspect there has been a similar culture shift in other children's hospitals. Furthermore, culture at our institution has allowed for short intervals of vasoactive infusions through a peripheral intravenous line which we believe is consistent with standard practice allowing for less reliance on CVL. In the literature there is little information on number of CVL insertions needed to demonstrate competency.

Arterial lines also showed a statistically significant decrease over time. We hypothesize this is due to increasing reliance on SpO_2_ compared to PaO_2_ in acute respiratory failure (21–23) and the ubiquitous presence of non-invasive blood pressure measurements. One study demonstrated a mean of 60 arterial line insertions to achieve 84% competency third attempt success rate for first year Anesthesiology Residents (9). As the pediatric patient population has much smaller vessels it is difficult to extrapolate this number to our population.

We believe our study has implications for our PCCM trainees. It is imperative for educators to be constantly reassessing the clinical landscape in an effort to make sure that trainees are receiving adequate educational experiences. PCCM is a growing field with a recent substantial increase in positions and applicants (24). As multiple studies have shown that there is a minimum number performed procedures required prior to obtaining competency, we need to decide if Fellowship trainees are receiving enough experience. Furthermore, if the number of procedures being performed by Fellowship trainees is decreasing, such a decrease could impair the ability of academic PCCM Attendings to maintain their procedural skills.

Our study has several limitations. First, this was a single-institution fellowship program, retrospectively performed study relying on electronic database entry which may have introduced errors and bias. Although, there was no significant change in the number of weeks Fellows spent on service nor the number of admissions per Fellow per year over the decade. Furthermore, while practice at one institution may or may not reflect changes on a national scale we believe the procedures being studied follow our hypothesis with regards to the changing national culture of PCCM described in the literature. During the 10-year period most of the Fellows completed a 3-year Fellowship; however, some left Fellowship early, others extended Fellowship for personal reasons, some stayed for a 4th year as a Cardiac Critical Care Fellow. To control for this, we used number of procedures per Fellow per year. Finally, we did not design the study to look directly at competence or number of attempts in completing the procedure as previously studied. Therefore, while it is possible that although there is a decreasing number of procedures per Fellow per year, the individual Fellow may still be performing enough to obtain competence. Unfortunately, our internal database does not record whether ultrasound was used for CVL or indirect laryngoscopy for intubations. Both of these technologies may alter the number of procedures to demonstrate competence and would be important to study.

We believe that this study has implications for future areas of investigation. We would hope that our findings could be corroborated at other institutions or in other databases. As a group we need to study how best to evaluate and improve competency in procedures. Possibilities include the use of additional operating room experience in the later years of Fellowship or the use of simulation to provide the needed skills. Simulation may be a key area to address the deficiency in number of procedures. Multiple studies have shown that simulation can effectively teach procedural skills (25–27). With regards to PCCM Fellowship trainees, Nishisaki et al. described an innovative “bootcamp” which showed that simulation can be used in Fellowship trainees to improve vascular access skills (28). Fleming et al. used compliance with a checklist as an assessment tool to evaluate PCCM Fellows in a simulated CVL placement which has given promise to help evaluate trainees (29).

## Conclusions

There has been a statistically significant decrease in the rate of central venous lines and arterial lines performed by PCCM Fellowship trainees per number of years in Fellowship over the last 10 years at a single institution, tertiary, academic, children's hospital. Since the literature describes a number of procedures which allow the learner to obtain competence if the current trend continues the decreasing number of procedures could lead to decreased competence in both Fellows and Attendings in PCCM. This observation has the potential for a significant impact on the education of our trainees and the safety of the patients that they care for.

## Data availability statement

The raw data supporting the conclusions of this manuscript will be made available by the authors, without undue reservation, to any qualified researcher.

## Author contributions

BE, CN, PR conceptualized and designed the study. BE, CN, MK, PR organized the database. MK performed the statistical analysis. BE, CN, MK, EB, RM, PR analyzed and interpreted the data. BE wrote the first draft of the manuscript. BE, CN, MK, EB, RM, PR drafted and revised the manuscript, are responsible for the reported research, and have approved the manuscript as submitted.

### Conflict of interest statement

The authors declare that the research was conducted in the absence of any commercial or financial relationships that could be construed as a potential conflict of interest.

## Abbreviations

ABP: American Board of Pediatrics
ACGME: Accreditation of Graduate Medical Education
CTICU: Cardiothoracic Intensive Care Unit
CVL: Central Venous Line
ICU: Intensive Care Unit
LMV: Length of Mechanical Ventilation
LOS: Length of Stay
PICU: Pediatric Intensive Care Unit
PCCM: Pediatric Critical Care Medicine
PRISM III: Pediatric risk of mortality
STAT: Society of Thoracic Surgeons-European Association for Cardio-Thoracic Surgery Scores
UN: Unknown.
